# Patient–clinician brain concordance underlies causal dynamics in nonverbal communication and negative affective expressivity

**DOI:** 10.1038/s41398-022-01810-7

**Published:** 2022-01-28

**Authors:** Dan-Mikael Ellingsen, Andrea Duggento, Kylie Isenburg, Changjin Jung, Jeungchan Lee, Jessica Gerber, Ishtiaq Mawla, Roberta Sclocco, Robert R. Edwards, John M. Kelley, Irving Kirsch, Ted J. Kaptchuk, Nicola Toschi, Vitaly Napadow

**Affiliations:** 1 Department of Psychology, University of Oslo, Oslo, Norway; 2 Department of Diagnostic Physics, Division of Radiology and Nuclear Medicine, Oslo University Hospital, Oslo, Norway; 3 Athinoula A. Martinos Center for Biomedical Imaging, Massachusetts General Hospital, Charlestown, MA USA; 4 Department of Biomedicine and Prevention, University of Rome ‘Tor Vergata’, Rome, Italy; 5 KM Fundamental Research Division, Korea Institute of Oriental Medicine, Daejeon, Korea (Republic of); 6 Department of Physical Medicine and Rehabilitation, Spaulding Rehabilitation Network, Harvard Medical School, Charlestown, MA USA; 7 Department of Radiology, Logan University, Chesterfield, MO USA; 8 Department of Anesthesiology, Brigham and Women’s Hospital, Boston, MA USA; 9 Endicott College, Beverly, MA USA; 10 Program in Placebo Studies & Therapeutic Encounter (PiPS), Beth Israel Deaconess Medical Center, Harvard Medical School, Boston, MA USA

**Keywords:** Neuroscience, Human behaviour

## Abstract

Patient–clinician concordance in behavior and brain activity has been proposed as a potential key mediator of mutual empathy and clinical rapport in the therapeutic encounter. However, the specific elements of patient–clinician communication that may support brain-to-brain concordance and therapeutic alliance are unknown. Here, we investigated how pain-related, directional facial communication between patients and clinicians is associated with brain-to-brain concordance. Patient–clinician dyads interacted in a pain-treatment context, during synchronous assessment of brain activity (fMRI hyperscanning) and online video transfer, enabling face-to-face social interaction. In-scanner videos were used for automated individual facial action unit (AU) time-series extraction. First, an interpretable machine-learning classifier of patients’ facial expressions, from an independent fMRI experiment, significantly distinguished moderately painful leg pressure from innocuous pressure stimuli. Next, we estimated neural-network causality of patient-to-clinician directional information flow of facial expressions during clinician-initiated treatment of patients’ evoked pain. We identified a leader–follower relationship in which patients predominantly led the facial communication while clinicians responded to patients’ expressions. Finally, analyses of dynamic brain-to-brain concordance showed that patients’ mid/posterior insular concordance with the clinicians’ anterior insula cortex, a region identified in previously published data from this study^1^, was associated with therapeutic alliance, and self-reported and objective (patient-to-clinician-directed causal influence) markers of negative-affect expressivity. These results suggest a role of patient-clinician concordance of the insula, a social-mirroring and salience-processing brain node, in mediating directional dynamics of pain-directed facial communication during therapeutic encounters.

## Introduction

The patient–clinician relationship can have a powerful impact on patient outcomes, across disorders [1, 2] and across therapies [3, 4]. The clinical encounter is particularly relevant across mental illnesses as well as in chronic psychosomatic conditions such as fibromyalgia, which is often characterized by uncertainty in diagnosis, treatment, and management [5], leading to challenges in building a therapeutic relationship between patient and clinician [6]. Difficulties in communication are commonly described as a major issue by both patients and clinicians, contributing to reduced medical adherence, suboptimal treatment, and poorer clinical outcomes [7–9]. Although research has identified nonverbal aspects of communication (e.g., pain-related facial expressions for chronic-pain patients) as important mediators of a therapeutic relationship, the causal dynamics of this mode of information transfer in clinical encounters is not well studied. Moreover, the brain processes supporting this mode of communication are unknown.

Nonverbal behavior such as facial expressions have been identified as central in clinical interactions, and better clinician accuracy in perceiving patient emotions is associated with a range of positive outcomes, including greater patient satisfaction, better treatment adherence, and improved comprehension and retention of medical information [10]. In fact, recent evolutionary frameworks have suggested that, beyond self-protection, a primary function of symptoms such as pain is to motivate expressions that will call for the attention of others who may be able to help [11]. However, expressions of pain vary depending on the social context, chronic or acute nature of the pain, and whether the individual feels safe [12, 13]. To date, most investigations of pain expressions have studied single individuals in isolation, a setting that may involve facial expression dynamics that are qualitatively different from those in actual social interactions. Thus, it is increasingly acknowledged that two-person (or more) studies during active interaction are needed in order to fully understand social processes [14]. This notion is especially pertinent for the study of physiological and brain processes underpinning social interactions [15]. Patient–clinician dynamics in nonverbal communication such as facial expressions [16], posture [17], and gestures [18] have been linked to empathy and clinical rapport, which may underpin dynamics in brain-to-brain coupling [19]. Similarly, in psychotherapy, reduced therapeutic alliance can reduce eye contact and physiological concordance between the patient and the therapist [20, 21].

Using two-person functional MRI (fMRI hyperscanning), we recently identified patient–clinician concordance in brain activity as a potentially key mechanism supporting therapeutic alliance in pain treatment [16]. Specifically, we found that patient–clinician dyads that had established a clinical relationship, relative to a control group, showed increased dynamic concordance in brain circuitry involved in theory-of-mind and social mirroring, such as the insula, temporoparietal junction (TPJ), and ventrolateral prefrontal cortex (vlPFC) [16]. Correspondingly, recent social neuroscience studies have shown brain-to-brain coupling in this circuitry during joint attention [22–24], mutual eye gaze [25], in addition to verbal communication (language comprehension) [26], and speaker–listener interaction [27]. Lateral prefrontal and insular cortices have also been associated with affective information flow during nonverbal communication using facial expressions [28]. These pioneering studies have spurred key questions about the causal directionality of facial expression transmission in the interacting patient/clinician dyad, and how these nonverbal communication dynamics may be associated with brain-to-brain concordance.

Here, we investigated the causal dynamics in patient–clinician facial communication in the context of pain treatment, using neural-network-based causality modeling of the dynamics of facial action units (AU). These AUs were algorithmically identified from videos of patients’ and clinicians’ faces during their interaction. Furthermore, we investigated whether these dynamics were associated with concordance in brain activity, using simultaneously recorded fMRI (see [16] for previously published data from this study). We hypothesized that pain-relevant patient–clinician facial communication would be associated with stronger brain concordance in circuitry implicated with social mirroring and theory-of-mind/empathy.

## Methods and materials

### Subjects

**Fig. 1:**
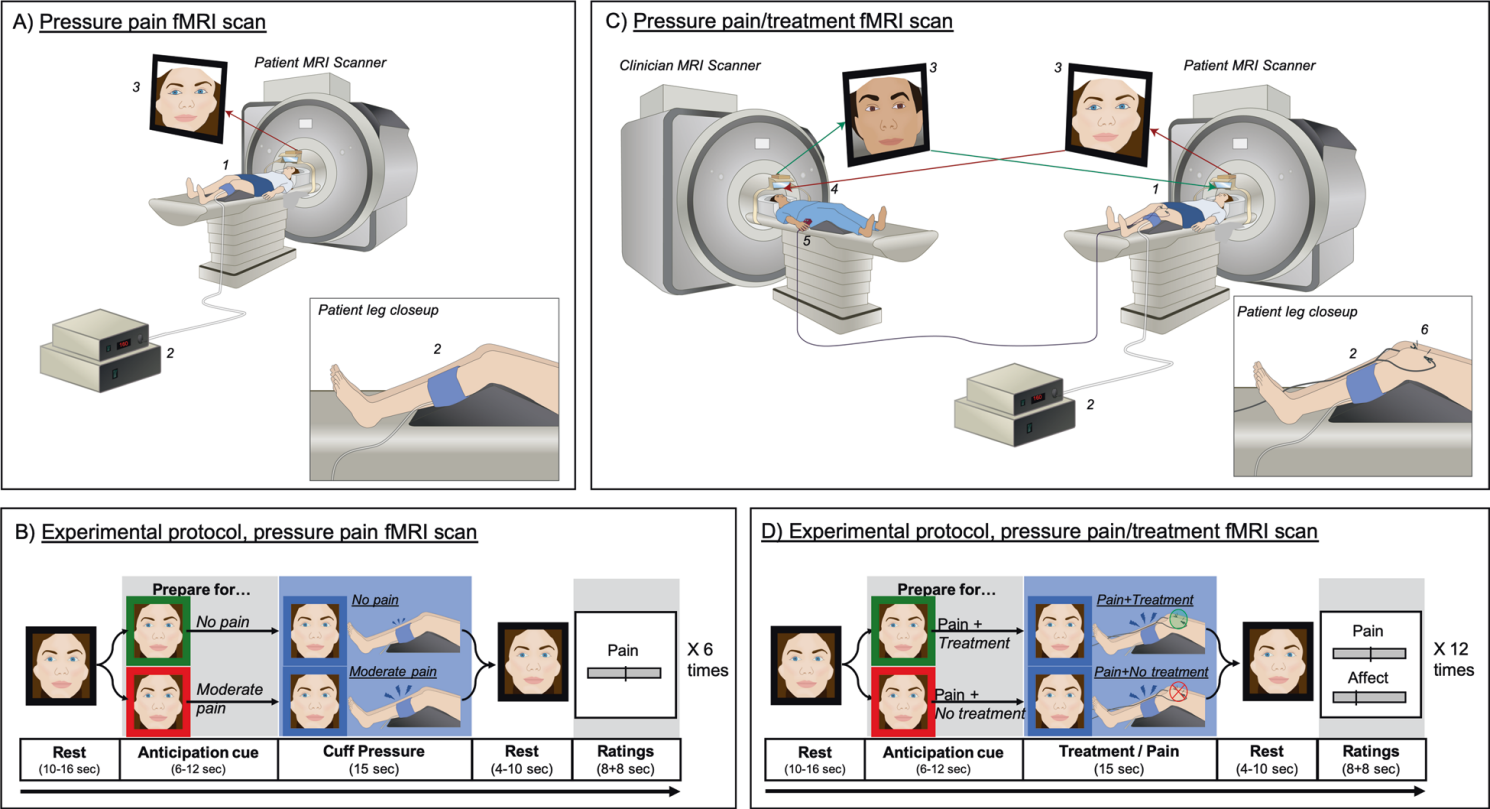
fMRI hyperscanning experimental environment and protocol. **A** In an initial “pain MRI”, the patient (1) received a series of moderately painful and nonpainful leg-cuff-pressure stimuli (2) while the clinician observed. Video recordings of patients’ facial expressions (3) during moderately and nonpainful cuff pressure were used in a machine learning classifier to identify facial expressions indicative of painful relative to nonpainful states. **B** Each pain MRI consisted of six repeated trials (three moderate pain, three no pain) in a pseudorandomized order. After a resting period, patients were shown a visual cue indicating whether the upcoming cuff pressure would be non-painful (green frame) or painful (red frame). Following this anticipation cue, patients received either innocuous (30 mmHg) or painful (individually calibrated to evoke moderate pain) cuff-pressure stimuli to their left leg. After each trial, patients rated pain intensity on a Visual Analog Scale. **C** In the subsequent “pain/treatment MRI”, the clinician (4) used a button box (5) to apply remotely activated subliminal electroacupuncture treatment (6) to the patient (1) while they received moderate cuff-pressure pain (2). In-scanner videos (3) were recorded and used for automated facial-feature extraction to investigate causal dynamics in nonverbal communication between the patient and the clinician, and their association with brain-activity concordance assessed with simultaneous fMRI. **D** Each pain/treatment MRI consisted of 12 trials (four verum, four sham, and four no treatment) in a pseudorandomized order. After a rest period (left), both participants were shown visual cues, indicating whether the next pain stimulus, applied to the patient, would be treated (green frame) or not treated (red frame) by the clinician (Anticipation phase). Next, patients received moderately painful cuff-pressure stimuli to their left leg, while clinicians applied or did not apply treatment, according to the preceding anticipation cue.

We enrolled 23 patients diagnosed with fibromyalgia (“patients”) and 22 licensed acupuncture practitioners (“clinicians”) to participate in an interactive pain-treatment experiment during fMRI hyperscanning. Participants were matched to form patient–clinician dyads (*n* = 40 total unique dyads), who were positioned in separate MRI scanners with nonverbal (i.e., facial expressions) communication enabled via online video connection (Fig. 1, see Supplementary Methods for full details).

The study was approved by the Massachusetts General Hospital institutional review board. All participants provided informed consent.

### Overall study protocol

At an initial behavioral visit, all patients were familiarized with the experimental protocol, provided informed consent, and went through a cuff-pressure pain-calibration procedure in order to select an individualized pressure level (mmHg) for evoking moderate pain (~40 out of 100, 0 = no pain, 100 = most pain imaginable). Painful deep-pressure stimuli were applied to the patients’ left lower leg using the Hokanson Rapid Cuff Inflator (Hokanson, Inc., Bellevue, WA, USA).

During the MRI visits, patients were first positioned in the MRI scanner. The clinician then entered and led the patient through the process of acupuncture needling, in which two needles were inserted above the patient’s left knee, proximal to the cuff, with MRI-safe electrodes attached to each needle. Following needle insertion, the clinician was positioned in the other MRI scanner, on the same floor in the same building. We applied a customized head-coil configuration, using a 64-channel head-coil bottom in combination with a small (4-channel) flex coil positioned over each subject’s forehead, in order to enable full facial coverage for video transfer. MRI-compatible video cameras enabled the participants to communicate nonverbally (e.g., eye movement and facial expressions) during the experimental hyperscanning runs. Each dyad completed two experiments, one in which the patient experienced moderately painful and nonpainful cuff-pressure stimulation while the clinician observed (pain MRI run, Fig. 1A, B), and another experiment in which the patient experienced pain while the clinician “treated” the patient’s pain with remotely controlled electroacupuncture (pain/treatment MRI run, Fig. 1C, D). Participants were informed that they could freely communicate their feelings nonverbally using facial expressions, provided they kept their head as still as possible (see Supplementary Methods for full details on the experimental protocol).

### Self-report assessments

#### Therapeutic alliance

The Consultation and Relational Empathy (CARE) questionnaire [29] was filled out by patients and clinicians, and used as a proxy for therapeutic alliance. The total scores for patient-rated CARE and clinician-rated CARE were calculated and averaged for each dyad to estimate dyad-wise therapeutic alliance.

#### Trait-affective expressivity

Patients filled out the Berkeley Expressivity Scale [30], as an assessment of individual (trait) tendency to express emotions and feelings. The scale has three subscales—negative expressivity, positive expressivity, and impulse strength, which are calculated as a sum of the items for each subscale.

### Video data processing

We recorded in-scanner videos from both scanners continuously during fMRI scan runs at a sampling rate of 20 frames per second, and used automated facial-feature extraction (Affectiva, Cambridge, MA) to obtain individual action-unit (AU) values for each video frame. Affectiva software extracts the extent of activation/movement of 20 facial AUs, such as brow raise/furrow, nose wrinkle, lip movement (raise, pucker, press, suck, and tighten), mouth open, chin raise, smirk, eye closure/widen, and jaw drop. A full list of AUs is available in Supplementary Table S1.

### Statistical analysis

#### Discrimination of pain states and ranking of facial-feature importance

The time courses of facial AUs extracted from patients’ facial video data during the pain MRI were used to train a nonlinear classifier, using the extreme gradient-boosting (XGBoost) algorithm [31], in order to (1) discriminate states of moderate pain from innocuous pressure based on patients’ facial data (Fig. S1A.1), and (2) extract the relative importance of each facial action unit for this classification (Fig. S1A.2). Precision and recall were also calculated. The contribution of each feature (i.e., facial action unit) to the final prediction performance of the model was evaluated and ranked by computing Shapley Additive explanations (SHAP) values [32].

#### Directed information flow of facial expressions between patients and clinicians

Video streams from patients and clinicians in the pain/treatment MRI were used to assess “information flow” (quantified using the general concept of Granger causality [33]) between the patient’s and clinician’s facial expressions (Figure S1B). Specifically, we investigated how the clinician’s facial expression affected the patient’s facial expression, and vice versa, using “Echo-State GC”, a GC implementation based on recurrent neural networks with minimal trainable parameter count [34, 35] (see Supplementary methods for details).

Granger causality was independently assessed during anticipation of pain and treatment/no treatment, which preceded pressure stimulus, consisting of 12 nonconsecutive data blocks for each dyad (6–12-seconds duration, pseudorandomized). Pain-related anticipation and expectancy constitute a key factor shaping pain outcomes [36–38] and are central to both pain phenomenology and patient–clinician interaction [39–41]. Thus, the pain/treatment anticipation phase provided an optimal window for causality analyses of clinically relevant nonverbal interaction while unimpeded by task-related differences in sensorimotor processes involved in evoked pain receipt (patients) and treatment application (clinicians). Furthermore, focusing on the anticipation phase aided inferences of how facial expression causality is associated with concordance in brain activity, which may be more confounded during differential nociceptive stimulation and motor activity for the patient and clinician. For every clinician–patient dyad, we estimated the GC strength for the time series of each of 20 facial action units in both directions, thus obtaining two (patient-to-clinician and clinician-to-patient) nonsymmetric 20×20 matrices of GC strength between face features, for each dyad and for each GC paradigm.

#### Statistical analysis of echo-state Granger causality estimates

To evaluate statistical significance of the patient-to-clinician and clinician-to-patient GC estimates, we constructed empirical null distributions for each paradigm using simulated “dyads” of subjects (patient–clinician dyads who did not actually interact), in order to minimize the presence of “pseudo-concordance” due to the shared structure of the experimental protocol. GC-estimate distributions for “real dyads” were contrasted with these null distributions using nonparametric statistics. Specifically, the causal link between any two signals was considered statistically significant if the median GC strength in the null distribution was significantly (*p* < 0.05) lower than the median GC strength across “real dyads” (Mann–Whitney U-test) (Fig. S1B.3). All the resulting p-values related to the GC matrices were then corrected for multiple comparisons using a false-discovery rate (FDR) correction procedure across all 400 matrix elements (alpha = 0.05).

#### BOLD fMRI acquisition, preprocessing, and analysis

See Supplementary Methods for details on MRI acquisition and preprocessing. To investigate whether dynamic brain-to-brain concordance in social-mirroring circuitry would be associated with patient–clinician facial expression communication related to pain, we calculated dynamic fMRI signal concordance for each patient–clinician dyad as previously reported [16]. After preprocessing of the fMRI data from the pain/treatment MRI scans, we performed two first-level GLM for each participant (one for each pain/treatment MRI run), using each trial anticipation phase as a separate regressor of interest (Fig. S1C.1). Rating periods and pain periods were also included in the GLM as regressors of no interest. This yielded 12 parameter estimate maps (one for each trial across both fMRI scan runs) for each individual. Next, we extracted mean Zstat scores within regions of interest (ROI) implicated in theory-of-mind processing and social mirroring, including the left anterior insula, the left vlPFC, and bilateral TPJ (see [16]) from each clinician (Fig. S1C.2), which were used as a trial-by-trial regressor in a second-level whole-brain regression GLM for time-synchronized fMRI data from their patient, and vice versa (Fig. S1C.3). This analysis produced a whole-brain map, for each individual, of regions that were dynamically coupled with their partner’s brain fMRI data throughout the interaction. These estimates were passed up to a group-level regression GLM, using FMRIB’s Local Analysis of Mixed Effects (FSL-FLAME1 + 2), where the influence of patient’s AU28 on the clinician’s facial expressions was used as a between-subject regressor (Fig. S1D). This facial feature was used as it (1) showed the highest-ranked influence in the machine-learning model trained to distinguish pain from innocuous pressure based on facial AU, and (2) because it exerted significant causal influence on the largest number of the clinicians’ AUs. We used the subjectwise overall causality strength of patients’ AU28 (lip sucking) on clinicians’ facial AUs (mean across all clinician AUs, first row in Fig. 3b) as the regressor of interest. Thus, the resulting group map indicated brain regions in which patients whose facial expressions influenced their clinician’s facial responses more strongly also showed greater dynamic coupling with their clinician’s social-mirroring circuitry. The results of this whole-brain analysis were corrected for multiple comparisons using FSL’s ‘cluster’ tool, which applies Gaussian Random Field theory to determine initial minimal extent threshold, using a voxel-wise cluster forming the statistical threshold of *z* = 2.3, and cluster significance threshold of *P* = 0.05 [42, 43]. This combination of FSL’s FLAME and a cluster threshold of *z* = 2.3 has been shown to be relatively conservative compared with other parametric approaches such as Ordinary Least Squares, especially for paradigms using moderate block lengths similar to our study, and yields a false error rate of approximately 5% [44].

## Results

### Therapeutic alliance

Ratings of therapeutic alliance (CARE) showed relatively high scores for both patients (mean ± SD: 42.20 ± 4.25, possible range: 9–45) and clinicians (mean ± SD: 35.69 ± 4.08), as previously reported [16]. Since we investigated both patient and clinician outcomes in facial expressions and brain activity, we calculated a mean value for the patient and clinician-rated CARE scores as an estimate for each dyad’s overall therapeutic alliance (mean ± SD: 39.26 ± 2.67).

### Classification of patients’ pain-related facial expression during clinician observation

**Fig. 2:**
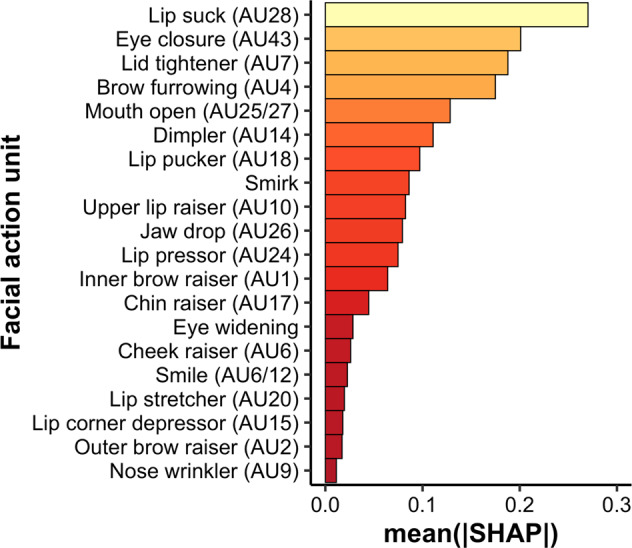
Unique importance of individual facial action units (AU) in classifying painful and nonpainful states. Following machine-learning classification, we calculated Shapley Additive explanations (SHAP) values, which indicate the relative contribution of each individual AU in distinguishing periods of painful leg pressure from periods of innocuous, nonpainful, leg pressure. AUs are numbered according to their corresponding Facial Action Coding System (FACS) code. The AUs “Smirk” and “Eye widening”, as estimated by the AFFDEX algorithm, do not correspond to specific AUs in the FACS framework.

The XGBoost model distinguished moderate pain from innocuous pressure conditions by employing the patient’s AU data yielding an AUC of 0.75, a precision of 72%, and a recall of 70% (Fig. 2a). This is comparable to previous studies using neuroimaging metrics to classify chronic pain from individuals without chronic pain [45]. SHAP estimation [46], reflecting the relative importance of each AU in distinguishing moderate pain from innocuous leg pressure (Fig. 2b), indicated that AU28 (lip sucking) yielded the largest contribution to the prediction of the pain state, followed by AU43 (eye closure), AU7 (tightening of the eyelids), and AU4 (brow furrowing), consistent with previous reports of facial expressions associated with pain [8, 47, 48].

### Causal influence of facial expression transfer between patients and clinicians

**Fig. 3:**
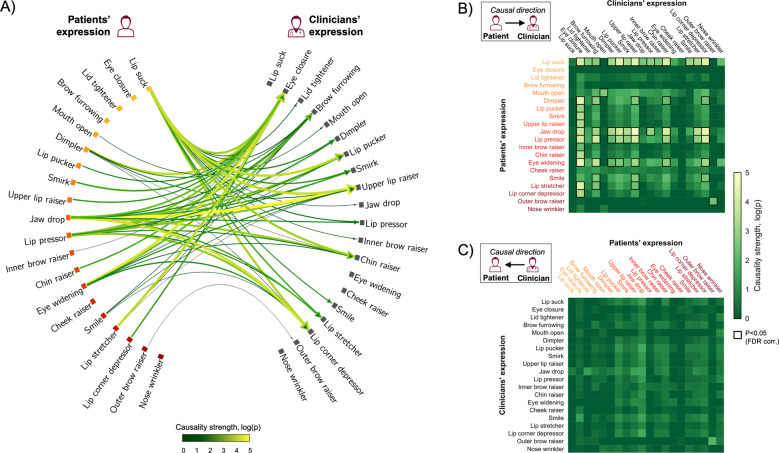
Median (across dyads) Granger causality strength between patients’ and clinicians’ facial action units (AUs) during pain treatment anticipation. **A** Significant causal links between patients’ AUs and clinicians’ AUs, surviving false discovery rate (FDR) correction for multiple comparisons, are shown with causal direction indicated by arrowheads and causality strength (log(*p*)) indicated by color and thickness. AUs are ordered according to SHAP values (the most influential AU at the top row) reflecting AU association with patients’ pain perception—i.e., importance in discriminating pain from innocuous pressure when no treatment was provided (i.e., bright yellow indicates a large, whereas dark red indicates a small, contribution to machine-learning-based prediction of patients’ pain vs. innocuous pressure, corresponding to the color shading in Fig. 2). Matrices (right) show causal links from the patient to the clinician (**B**) and from the clinician to the patient (**C**). Same as for the connectogram (**A**), AUs are ordered according to their ranked contribution to the independent classification of pain for nonpain states (bright yellow indicates large, while dark red indicates small contribution). Each cell in the matrices shows the corresponding Granger causality strength. The causal links that survived FDR correction for multiple comparisons across all possible matrix cells are highlighted by black rectangles. Patients’ “lip suck”, which showed the strongest unique contribution to the discrimination of pain states, was also the AU that influenced the dynamics of the largest number of facial AU in the clinician when treating evoked cuff-pressure pain (**B**). No significant causal links were found in the opposite direction—i.e., between clinicians’ facial AUs and patients’ AUs (**C**).

Granger causality analyses of facial expressions during anticipation of evoked pain/treatment indicated a strong asymmetry in which patients’ facial expressions caused a range of facial expressions displayed by the clinician, while clinician AUs showed minimal causal influence on patient expressions (Fig. 3). The median influence (i.e., Granger causality strength) of the variability in individual patient AUs on the variability of the clinician AUs was significantly higher compared with the median of the estimated null distribution. Specifically, 59 causal links survived false discovery rate (FDR, *α* = 0.05) correction for multiple comparisons of patient-to-clinician directed causality, while there were no significant causal links for clinician-to-patient directed causality after FDR correction. Notably, the dynamics in patients’ AU28 (lip sucking), which was also identified as the feature that best discriminated between moderate pain and innocuous pressure, exerted a significant causal influence on the dynamics of the largest number of clinician AUs. Conversely, clinicians’ AU43 (eye closure), which was the second most discriminative feature of patients’ perceived pain, was the facial feature most influenced by the patients’ facial expressions (i.e., causally influenced by the highest number of patient AUs). The corresponding exploratory analyses of Granger causality during the pain phase showed a similar pattern, with a strong unidirectional effect of patient-to-clinician causality for a wide range of AUs, but no significant causal links in the opposite direction (Fig. S2).

### The causal strength of patients’ AU28 on clinicians’ facial expressions is related to patients’ dynamic brain-activity concordance with clinicians’ anterior insula

**Fig. 4:**
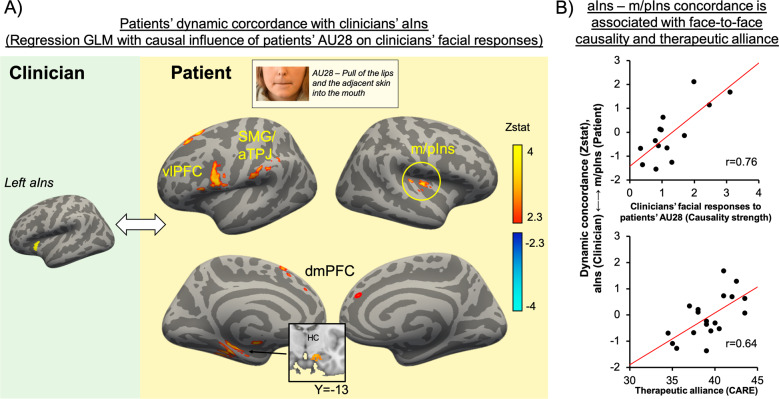
Association between facial-expression exchange dynamics and brain-to-brain concordance during pain-treatment anticipation. (**A**) A voxelwise GLM regression indicated that the causal strength of patients’ AU28 on clinicians’ facial responses overall (across all clinician AUs) was associated with dynamic brain concordance between clinicians’ aINS activity and patients’ fMRI activity in the m/pIns, vlPFC, SMG/aTPJ, dmPFC, and the HC. (**B**) Furthermore, dyads characterized by greater aIns (clinician) to m/pIns (patient) concordance also reported greater therapeutic alliance at the preceding clinical intake (Pearson’s *r* = 0.64, *p* = 0.014). aIns: anterior insula; vlPFC: ventrolateral prefrontal cortex; SMG: supramarginal gyrus; aTPJ: anterior temporoparietal junction; m/pIns: middle/posterior insula; dmPFC: dorsomedial prefrontal cortex; HC: hippocampus; AU28: action unit 28; CARE: Consultation and relational empathy questionnaire.

A whole-brain regression analysis was performed to investigate the degree to which causal influence of patients’ pain-related facial expressions on clinicians’ facial responses would be associated with dynamic brain concordance in social-mirroring circuitry. The results indicated that, for dyads in which clinicians’ facial expressions were more strongly influenced (i.e., higher causal strength) by patients’ AU28 dynamics, greater dynamic brain concordance was also noted between clinicians’ aIns activity and patients’ fMRI activity in middle/posterior insula (m/pIns), dorsomedial (dmPFC) and ventrolateral prefrontal cortex (vlPFC), supramarginal gyrus (SMG)/anterior temporoparietal junction (aTPJ), and hippocampus (HC) (Fig. 4a).

Next, we evaluated if the dynamic brain concordance noted above was also associated with clinically relevant outcomes. We found that dynamic concordance between clinicians’ aINS and patients’ m/pIns (extracted mean Zstat values from each dyad) was significantly associated with therapeutic alliance (CARE scores, averaged between patient-rated and clinician-rated scores, *r* = 0.64, *p* = 0.01), suggesting that dyads with higher therapeutic alliance showed stronger insular dynamic concordance during pain-treatment anticipation (Fig. 4b).

### Insula cortex dynamic brain-to-brain concordance is positively associated with patients’ trait (negative) expressiveness

**Fig. 5:**
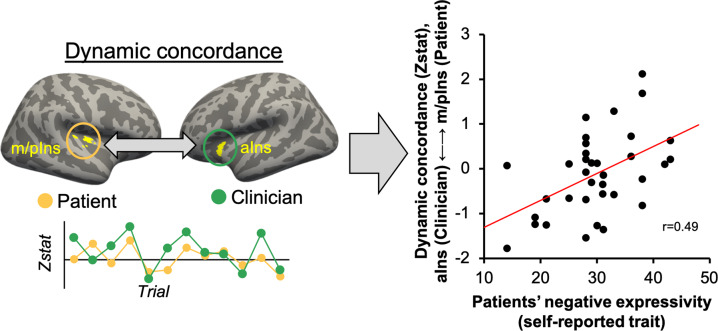
Association between brain-to-brain insula concordance and patients’ tendency to express negative affect. Dynamic brain-to-brain concordance between aIns (clinicians) and m/pIns (patients) was significantly associated with patients’ self-reported trait-negative expressivity (Berkeley Expressivity Questionnaire) (Pearson’s *r* = 0.49, *p* = 0.002). Thus, dynamic concordance was greater in dyads in which patients showed a higher tendency to express negative affect. m/pIns: mid/posterior insula; aIns: anterior insula.

Next, we investigated whether dynamic brain concordance was also associated with personality factors—i.e., patients’ trait expressivity of negative affect. A Pearson correlation coefficient indicated that dynamic concordance between clinicians’ aINS and patients’ m/pIns was significantly associated with patients’ negative expressivity, assessed by the Berkeley Expressivity Scale (*r* = 0.49, *p* = 0.002, Fig. 5) [49]. Note that this analysis was performed on a larger sample (*n* = 37 dyads), as intact fMRI and questionnaire data existed for more dyads than those with intact facial video data. However, the same analysis shows a corresponding effect when performed only on dyads with intact facial data for both participants (*r* = 0.62, *p* = 0.018).

## Discussion

In this study, we combined patient–clinician simultaneous fMRI (hyperscanning) with facial expression recording and neural-network-based Granger causality analyses to investigate the causal dynamics of facial expressions in a therapeutic interaction, and how these dynamics are supported by patient–clinician concordance in brain activity. Using automated extraction of facial action units (AUs) from video time series of patients’ and clinicians’ faces during social interaction, we first applied a nonlinear machine-learning classifier, which predicted whether or not patients were experiencing pain based on AU data alone. Next, Granger causality analyses indicated that patients’ facial expressions caused a range of facial responses by clinicians, but not vice versa, suggesting a leader–follower relationship in which patients led the facial communication while clinicians responded. We then demonstrated that the most salient causal relationship—clinicians’ global facial expression response to patients’ AU28, which exerted the highest relative influence on the machine learning classifier outcomes—was also associated with patient–clinician dynamic brain concordance in circuitry implicated in social mirroring and theory-of-mind. Specifically, the strength of patient-to-clinician directed causality was positively associated with patients’ vlPFC, SMG/aTPJ, and mid/posterior insula concordance with clinicians’ fMRI response in the anterior insula. Finally, insula-to-insula concordance in particular was associated with self-reported therapeutic alliance and patients’ trait expressivity, suggesting that patients with a higher tendency to express negative emotions also showed stronger insula-to-insula concordance with their clinician.

Expression of pain is highly dependent on social context and the state of the individual [11]. For example, patients with chronic pain often have very different communicative motivations than individuals without chronic pain experiencing an acute painful situation. Expression of pain may also differ, depending on whether it is expressed in a social context or alone [12]. Many of the studies investigating pain facial expressions have studied individuals in isolation. Here, we studied patient–clinician pairs during a therapeutic interaction, which provided a qualitatively different context compared with single-subject studies or studies of healthy volunteers roleplaying as patient and clinician. Hence, notwithstanding the inherent limitations of the MRI environment, our protocol design included unique elements, which may have increased ecological validity, leading to clinically relevant associations between psychosocial behavioral and neural processing.

Our pain-expression model, using the state-of-the art method to assign univocal importance values to features influencing classification (SHAP), identified several facial AUs consistent with previous reports of pain-related expressions, such as eye closure (AU43), lid tighten (AU7), brow furrow/lowerer (AU4), and mouth open (AU52) [8, 47, 48]. Notably, a recent systematic review identified a relatively consistent set of AUs associated with experimental and clinical pain, except for eye closure, which was more prominent for clinical pain [47]. Since complete eye closure involves the activation of the orbicularis oculi muscle, which is also activated—albeit less intensely—during lid-tightening (AU7) and cheek-raise (AU6), the authors speculated that eye closure may signal more severe, or prolonged, pain in the context of chronic pain. In our study, eye closure ranked as the second most influential AU for classifying experimentally evoked pressure pain relative to innocuous pressure—greater than lid-tighten and cheek-raise—which may reflect the patients’ chronic-pain condition. One notable exception in our results was that wrinkling of the nose (AU9), which is a relatively commonly reported AU for pain, did not strongly contribute to evoked pain in our study.

Interestingly, the AU that had the strongest influence on classifying pain from innocuous pressure in our machine-learning model was lip sucking (AU28). Moreover, lip sucking, when expressed by the patient, was also the AU that exerted significant causal influence on the highest number of clinician AUs during the pain/treatment MRI, suggesting that this patient expression most readily influenced clinicians’ facial expression. Although not necessarily included as part of a “canonical” pain expression, some studies have indeed associated AU28 with pain [8, 48]. Furthermore, artificial-intelligence algorithms that estimate composite emotions from patterns of individual AUs found that lip-sucking dynamics influences the likelihood of negative affect (i.e., anger, sadness, and disgust) but not positive affect [50]. In the context of pain treatment in our study, dynamics in lip sucking may thus have conveyed negative affect associated with current or upcoming pain, from patient to clinician.

Granger causality analyses of facial-expression time series (based on nonlinear neural networks with minimal parameter count and hence little-to-no overfitting), showed a robust asymmetry in which patient expressions caused a wide range of facial responses by the clinician, but not vice versa. One possibility is that clinicians, in response to patients’ expressions, were attempting to use nonverbal communication as a means for consolation or expressing support for their patient, even though patients were less responsive to expressions by the clinician. Importantly, we applied causality-estimation modeling based on nonlinear neural networks, which has been shown to be superior to traditional Granger causality methods in estimating highly nonlinear, directed coupling in complex networks such as an ensemble of facial AUs from distinct individuals.

We previously found that treatment-related change in mirroring of facial expressions was associated with therapeutic alliance and patient analgesia [16]. This raises the question of whether patients and clinicians show causal directionality consistent with mirroring overall at the level of individual AUs. Notably, our Granger causality analyses, across both treatment and no-treatment trials, showed significant influence of the patients’ opening of the mouth (AU25/26/27) and raising of the (outer) eye brow (AU20) on the clinicians’ facial activation of these same AUs, but not similar symmetric causality for other AUs. Thus, aside from the former AUs, our results do not suggest a simple overall AU-to-AU mirroring pattern. However, patients’ expressions caused robust expression of clinician AUs that were highly associated with pain states for the patients, such as eye closure (AU43) and brow furrow (AU4). In fact, among all of the clinician AUs that were significantly influenced by patient expressions, 40 causal links (67.8%) were among the ten AUs independently identified as most strongly influencing the machine-learning classifier trained to discriminate painful states from nonpainful states. Furthermore, considering both patient and clinician expressions, the results showed 21 significant connections among the ten AUs most associated with pain (Fig. 3b, top-left quadrant), compared with only seven causal links among the ten AUs least associated with pain (Fig. 3b, bottom-left quadrant). This may indicate that clinicians responded to patients’ expressions (in general, but particularly for AUs associated with pain) by making pain-related facial expressions, which may be reflective of mirroring in a broader sense. Specifically, rather than strict “mimicry” of individual AUs, clinician mirroring of patients’ expressions may have been characterized by more flexible constellations of pain-related expressions. This is consistent with previous research suggesting that individualized pain-related facial expressions vary flexibly along higher-level patterns of AUs rather than expressing a uniform (prototypical) set of individual AUs [51]. It has also been proposed that it may be functionally meaningful to classify pain expressions more broadly into unintentional, reflexive expressions vs. intentional, reflective expressions following a cognitive process. This hypothesis is supported by observers’ tendency to respond to pain expressions with facial displays of more immediate “visceral” emotions vs. more “controlled” expressions, respectively, reflecting higher-level conceptual mirroring [52].

Using hyperscanning fMRI, we tested whether patient-to-clinician-directed information flow in facial expressions was associated with dynamic brain-to-brain concordance. For dyads showing stronger causal influence of patients’ AU28 (lip sucking) on clinicians’ (global) facial expressions, patients showed stronger dynamic concordance with the clinician’s anterior insula (aIns). The anterior and mid-sections of the insula, a key hub of the salience network, play a fundamental role in integrating sensory signals with cognitive and affective information in order to guide behavioral decisions [53]. Furthermore, multiple studies have indicated that the aIns, along with the vlPFC, dlPFC, and TPJ, constitutes a network implicated in social processes such as theory-of-mind and empathy [54, 55]. Moreover, the aIns is thought to be part of an extended mirror neuron network [56, 57]. Our results demonstrate that patients who exerted stronger causal influence on clinicians’ facial expressions, showed higher concordance, with the clinician’s aIns, in circuitry also implicated in social processing, such as the mid/posterior insula (m/pIns), ventrolateral prefrontal cortex (vlPFC), and a region of the supramarginal gyrus overlapping with the anterior TPJ. A previous two-person fMRI study of nonverbal communication [28] reported brain-to-brain coupling between a “sender” and “perceiver” of facial expressions in a set of regions, including the insula, ventro-/dorsolateral PFC, precuneus, and hippocampus. In addition, a functional near infrared spectroscopy (fNIRS) hyperscanning study reported increased facial expressivity and eye gaze, which was underpinned by activation and cross-brain coupling of vlPFC, dlPFC, and the TPJ, when participants were sharing personal information interactively, relative to a nonsharing condition [58]. These studies support the importance of such social-mirroring circuitry to interpersonal interactions.

Previous studies have also linked activation of this circuitry with pain expressions directly. One study found that pain expressions in response to experimental pain were associated with activation of anterior and posterior insula along with somatosensory areas and midline circuitry (posterior and pregenual anterior cingulate cortices) [59]. Another study found that for patients with chronic back pain, relative to healthy controls, pain-related facial expressions were robustly associated with trial-by-trial fMRI dynamics in a range of brain regions, including lateral prefrontal and insular cortices, precuneus, and primary motor areas [60].

Notably, in our study, concordance between clinicians’ aIns and patients’ m/pIns fMRI response also showed a significant association with scores on the CARE questionnaire, such that patient–clinician dyads with higher insula cortex concordance reported higher therapeutic alliance. Furthermore, patients that showed stronger insula-to-insula dynamic concordance with the clinician also reported higher trait tendency to express negative affect, as assessed by the Berkeley Expressivity Questionnaire (note that this finding was consistent for both the full sample and for the subsample with intact facial-expression video data). Both evolutionary theoretical frameworks [61] and computational models in cognitive science [51] suggest that amplified expressivity of negative affect and pain may be utilized as part of a successful behavioral strategy for eliciting altruistic behavior by peers. Our findings suggest that brain-to-brain insula concordance may underlie facial communication dynamics driven by patients’ expressivity of negative affect.

Our study has several limitations. First, due to the limited sample of intact facial-expression data, our study was likely underpowered, which increases the risk of type-II errors. Thus, the observed classification accuracy (72%) for pain relative to nonpain states, based on facial AU time courses, may be improved with additional training data. Indeed, a recent study, using a deep-learning approach with a larger training data set of static images of facial expressions associated with shoulder pain, reported higher accuracy [62], suggesting that facial-expression data have appropriate information value for classifying pain from nonpain. Furthermore, while participants in this study were real clinicians and real patients with chronic pain, the MRI environment is inherently limited in terms of ecological validity due to the supine position of participants and the inability to communicate verbally during the pain/treatment fMRI scan. Future studies may investigate facial communication and brain-to-brain concordance using electroencephalography [63] or near-infrared spectroscopy [58], which would enable a more naturalistic patient–clinician interaction with more mobility, though at the cost of lower spatial resolution and depth. Finally, the clinical context in this study may not generalize to other kinds of patient–clinician interactions (e.g., psychotherapy, general medicine, and physical therapy).

In conclusion, we found that patients’ facial expressions during pain treatment had a strong dynamically causal effect on the clinicians’ facial expressions, but not vice versa. Furthermore, we found that patient–clinician concordance in insula-cortex activity was positively associated with larger causal influence of patients’ pain-related expressions on clinicians’ facial responses. Our findings specify brain-behavioral dynamics that may be central to successful patient–clinician interactions.

## Supplementary information

Supplementary information

## References

[1] Kaptchuk TJ, Kelley JM, Conboy LA, Davis RB, Kerr CE, Jacobson EE (2008). Components of placebo effect: randomised controlled trial in patients with irritable bowel syndrome. BMJ.

[2] Blasi ZD, Harkness E, Ernst E, Georgiou A, Kleijnen J (2001). Influence of context effects on health outcomes: a systematic review. Lancet.

[3] Ferreira PH, Ferreira ML, Maher CG, Refshauge KM, Latimer J, Adams RD (2013). The therapeutic alliance between clinicians and patients predicts outcome in chronic low back pain. Phys Ther.

[4] Wampold BE (2015). How important are the common factors in psychotherapy? An update. World Psychiatry.

[5] Clauw DJ (2014). Fibromyalgia: a clinical review. JAMA.

[6] Chen AT, Swaminathan A (2020). Factors in the building of effective patient-provider relationships in the context of fibromyalgia. Pain Med.

[7] Henry SG, Matthias MS (2018). Patient-clinician communication about pain: a conceptual model and narrative review. Pain Med.

[8] Ruben MA, Hall JA (2016). A lens model approach to the communication of pain. Health Commun.

[9] Butow P, Sharpe L (2013). The impact of communication on adherence in pain management. Pain.

[10] Hall JA (2011). Clinicians’ accuracy in perceiving patients: Its relevance for clinical practice and a narrative review of methods and correlates. Patient Educ Counseling.

[11] Steinkopf L (2016). An evolutionary perspective on pain communication. Evolut Psychol.

[12] Karmann AJ, Lautenbacher S, Bauer F, Kunz M (2014). The influence of communicative relations on facial responses to pain: does it matter who is watching?. Pain Res Manag.

[13] Karos K, Meulders A, Goubert L, Vlaeyen JWS (2020). Hide your pain: social threat increases pain reports and aggression, but reduces facial pain expression and empathy. J Pain.

[14] Christov-Moore L, Iacoboni M. Emotions in interaction: toward a supraindividual study of empathy. In: Martinovsky B (ed). *Emotion in Group Decision and Negotiation*. Dordrecht, Netherlands: Springer; 2015. pp 1–32.

[15] Redcay E, Schilbach L (2019). Using second-person neuroscience to elucidate the mechanisms of social interaction. Nat Rev Neurosci.

[16] Ellingsen D-M, Isenburg K, Jung C, Lee J, Gerber J, Mawla I (2020). Dynamic brain-to-brain concordance and behavioral mirroring as a mechanism of the patient-clinician interaction. Sci Adv.

[17] Ramseyer F, Tschacher W (2011). Nonverbal synchrony in psychotherapy: Coordinated body movement reflects relationship quality and outcome. J Consult Clin Psychol.

[18] Tschacher W, Rees GM, Ramseyer F (2014). Nonverbal synchrony and affect in dyadic interactions. Front Psychol.

[19] Koban L, Ramamoorthy A, Konvalinka I (2019). Why do we fall into sync with others? Interpersonal synchronization and the brain’s optimization principle. Soc Neurosci.

[20] Marci CD, Orr SP (2006). The effect of emotional distance on psychophysiologic concordance and perceived empathy between patient and interviewer. Appl Psychophysiol Biofeedback.

[21] Marci CD, Ham J, Moran E, Orr SP (2007). Physiologic correlates of perceived therapist empathy and social-emotional process during psychotherapy. J Nerv Ment Dis.

[22] Bilek E, Ruf M, Schäfer A, Akdeniz C, Calhoun VD, Schmahl C (2015). Information flow between interacting human brains: Identification, validation, and relationship to social expertise. Proc Natl Acad Sci USA.

[23] Redcay E, Dodell-Feder D, Pearrow MJ, Mavros PL, Kleiner M, Gabrieli JD (2010). Live face-to-face interaction during fMRI: a new tool for social cognitive neuroscience. Neuroimage.

[24] Bilek E, Stößel G, Schäfer A, Clement L, Ruf M, Robnik L (2017). State-dependent cross-brain information flow in borderline personality disorder. JAMA Psychiatry.

[25] Koike T, Tanabe HC, Okazaki S, Nakagawa E, Sasaki AT, Shimada K (2016). Neural substrates of shared attention as social memory: A hyperscanning functional magnetic resonance imaging study. NeuroImage.

[26] Silbert LJ, Honey CJ, Simony E, Poeppel D, Hasson U (2014). Coupled neural systems underlie the production and comprehension of naturalistic narrative speech. Proc Natl Acad Sci USA.

[27] Stephens GJ, Silbert LJ, Hasson U (2010). Speaker-listener neural coupling underlies successful communication. Proc Natl Acad Sci USA.

[28] Anders S, Heinzle J, Weiskopf N, Ethofer T, Haynes J-D (2011). Flow of affective information between communicating brains. NeuroImage.

[29] Mercer SW, Maxwell M, Heaney D, Watt GC (2004). The consultation and relational empathy (CARE) measure: development and preliminary validation and reliability of an empathy-based consultation process measure. Fam Pr.

[30] Gross JJ, John OP (1997). Revealing feelings: facets of emotional expressivity in self-reports, peer ratings, and behavior. J Personal Soc Psychol.

[31] Chen T, Guestrin C. XGBoost: A Scalable Tree Boosting System. In: *Proceedings of the 22nd ACM SIGKDD International Conference on Knowledge Discovery and Data Mining*. New York, NY, USA: Association for Computing Machinery; 2016. 785–94.

[32] Štrumbelj E, Kononenko I (2014). Explaining prediction models and individual predictions with feature contributions. Knowl Inf Syst.

[33] Granger CWJ (1969). Investigating causal relations by econometric models and cross-spectral methods. Econometrica.

[34] Duggento A, Guerrisi M, Toschi N. Recurrent neural networks for reconstructing complex directed brain connectivity. In: *2019 41st Annual International Conference of the IEEE Engineering in Medicine and Biology Society (EMBC)*. IEEE; 2019. pp 6418–21. 10.1109/EMBC.2019.8856721 31947311

[35] Duggento A, Guerrisi M, Toschi N. Echo state network models for nonlinear granger causality. Philos Trans Royal Soc. 2021;379:20200256. 10.1098/rsta.2020.0256 34689621

[36] Ellingsen DM, Wessberg J, Eikemo M, Liljencrantz J, Endestad T, Olausson H (2013). Placebo improves pleasure and pain through opposite modulation of sensory processing. Proc Natl Acad Sci USA.

[37] Wager TD, Rilling JK, Smith EE, Sokolik A, Casey KL, Davidson RJ (2004). Placebo-induced changes in FMRI in the anticipation and experience of pain. Science.

[38] Palermo S, Benedetti F, Costa T, Amanzio M (2015). Pain anticipation: An activation likelihood estimation meta-analysis of brain imaging studies. Hum brain Mapp.

[39] Chen P-HA, Cheong JH, Jolly E, Elhence H, Wager TD, Chang LJ (2019). Socially transmitted placebo effects. Nat Hum Behav.

[40] Wager TD, Atlas LY (2015). The neuroscience of placebo effects: connecting context, learning and health. Nat Rev Neurosci.

[41] Kaptchuk TJ, Hemond CC, Miller FG (2020). Placebos in chronic pain: evidence, theory, ethics, and use in clinical practice. BMJ.

[42] Beckmann CF, Jenkinson M, Smith SM (2003). General multilevel linear modeling for group analysis in FMRI. NeuroImage.

[43] Woolrich MW, Behrens TEJ, Beckmann CF, Jenkinson M, Smith SM (2004). Multilevel linear modelling for FMRI group analysis using Bayesian inference. NeuroImage.

[44] Eklund A, Nichols TE, Knutsson H (2016). Cluster failure: Why fMRI inferences for spatial extent have inflated false-positive rates. Proc Natl Acad Sci USA.

[45] Torrado-Carvajal A, Toschi N, Albrecht DS, Chang K, Akeju O, Kim M (2021). Thalamic neuroinflammation as a reproducible and discriminating signature for chronic low back pain. Pain.

[46] Lundberg SM, Lee S-I. A Unified Approach to Interpreting Model Predictions. Adv Neural Inf Process Syst. 2017; 30.

[47] Kunz M, Meixner D, Lautenbacher S (2019). Facial muscle movements encoding pain—a systematic review. Pain.

[48] Larochette A-C, Chambers CT, Craig KD (2006). Genuine, suppressed and faked facial expressions of pain in children. Pain.

[49] Gross JJ, John OP (1995). Facets of emotional expressivity: three self-report factors and their correlates. Pers Individ Differ.

[50] McDuff D, Mahmoud A, Mavadati M, Amr M, Turcot J, Kaliouby R et al. AFFDEX SDK: A cross-platform real-time multi-face expression recognition toolkit. In: *Proceedings of the 2016 CHI Conference Extended Abstracts on Human Factors in Computing Systems*. San Jose California USA: ACM; 2016, pp 3723–6.

[51] Kunz M, Lautenbacher S (2014). The faces of pain: A cluster analysis of individual differences in facial activity patterns of pain. Eur J Pain.

[52] Craig KD, Versloot J, Goubert L, Vervoort T, Crombez G (2010). Perceiving pain in others: automatic and controlled mechanisms. J Pain.

[53] Craig AD (2009). How do you feel–now? The anterior insula and human awareness. Nat Rev Neurosci.

[54] Bernhardt BC, Singer T (2012). The neural basis of empathy. Annu Rev Neurosci.

[55] Schurz M, Radua J, Tholen MG, Maliske L, Margulies DS, Mars RB. et al. Toward a hierarchical model of social cognition: A neuroimaging meta-analysis and integrative review of empathy and theory of mind. Psychol Bull. 2020. 10.1037/bul0000303. 10.1037/bul0000303 33151703

[56] Schippers MB, Roebroeck A, Renken R, Nanetti L, Keysers C (2010). Mapping the information flow from one brain to another during gestural communication. Proc Natl Acad Sci USA.

[57] Iacoboni M (2009). Imitation, empathy, and mirror neurons. Annu Rev Psychol.

[58] Cañigueral R, Zhang X, Noah JA, Tachtsidis I, Hamilton AF, de C (2021). Facial and neural mechanisms during interactive disclosure of biographical information. NeuroImage.

[59] Kunz M, Chen J-I, Lautenbacher S, Vachon-Presseau E, Rainville P (2011). Cerebral regulation of facial expressions of pain. J Neurosci.

[60] Vachon-Presseau E, Roy M, Woo C-W, Kunz M, Martel M-O, Sullivan MJ (2016). Multiple faces of pain: effects of chronic pain on the brain regulation of facial expression. Pain.

[61] Steinkopf L (2017). Disgust, empathy, and care of the sick: an evolutionary perspective. Evol Psychol Sci.

[62] Bargshady G, Zhou X, Deo RC, Soar J, Whittaker F, Wang H (2020). Enhanced deep learning algorithm development to detect pain intensity from facial expression images. Expert Syst Appl.

[63] Anzolin A, Isenburg K, Grahl A, Toppi J, Yücel M, Ellingsen DM et al. Patient-clinician brain response during clinical encounter and pain treatment. In: IEEE Engineering in Medicine and Biology Society. IEEE; 2020. 10.1109/EMBC44109.2020.9175608 PMC8096120 33018278

