# Supplementary material for: Patient–clinician brain concordance underlies causal dynamics in nonverbal communication and negative affective expressivity
Source: Transl Psychiatry. 2022 Jan 28;12:44. doi: 10.1038/s41398-022-01810-7 (PMC8799700; doi:10.1038/s41398-022-01810-7)
Supplement: Supplementary file 1 — Supplementary information [file 41398_2022_1810_MOESM1_ESM.pdf]

Supplemental information

Supplementary Methods and Materials

Subjects

23 patients diagnosed with fibromyalgia (Wolfe et al. [1] criteria) for at least 1 year were enrolled (Age: 39.95±10.93 (Mean±SD); all female; Race/Ethnicity: 18 Caucasian, 2 Hispanic, 2 African-American, 1 multiracial). We also enrolled 22 licensed acupuncturists, who had completed at minimum a 3-year Masters-level program, or were in their final year of training and completing clinical internships (Age: 44.32±12.81; 15 female; Race/Ethnicity: 18 Caucasian, 1 Hispanic, 1 African-American, 1 Asian, 1 multiracial). All participants received monetary compensation for their participation. Each participant was assigned to complete two MRI visits (with and without a prior clinical interaction, see below), each with a different partner in order to avoid carryover effects due to the initial relationship. 20 patients and 20 clinicians participated for at least 1 MRI visit. Of these, 3 patients (2 due to scheduling issues, 1 due to claustrophobia) and 3 clinicians (2 due to scheduling issues, 1 due to scanner discomfort) dropped out after 1 MRI visit.

Although hyperscanning was completed on a total of 40 unique dyads, the scans of 2 dyads were incomplete due to scanner malfunction, and 1 scan was incomplete due to patient withdrawal following claustrophobia mid-scan, resulting in a final sample of 37 unique dyads, which were included in analyses of MRI data without facial data. MRI visits were conducted either after the clinician had performed a clinical intake with the patient (Clinical-Interaction MRI) or without such a prior intake (No-Interaction MRI, counterbalanced order), but were otherwise identical in nature. The purpose of the intake was to increase the dynamic range in

the rated patient-clinician therapeutic alliance prior to the MRI (See [2] for more comprehensive details on the clinical intake). However, due to the complex study structure of fMRI hyperscanning with online video transfer of full-facial view between scanners, we were not able to obtain video recordings of sufficient quality for artificial intelligence (AI)–based facial recognition and processing from all dyads. Complete and intact face video data were available for 21 individual patient datasets and 24 clinician datasets, resulting in 14 dyads with intact data for both the patient and the clinician. Due to this limited sample, data processing and statistical analyses were not pursued for contrasts between Clinical-Interaction vs. No-Interaction conditions.

Due to the lack of previous data on dynamic concordance, we could not estimate power using dyad-based metrics. Our pilot data from clinicians applying treatment for the evoked heat pain of a 'patient' confederate[3], we found a mean Blood oxygen level-dependent (BOLD) percent change (within-subjects) for 'treatment' relative to 'no treatment' of 1.25±1.53 (mean±SD). An a priori power analysis (paired, two-tailed, α=0.05) indicated that 15 subjects would be required for 85% power to detect this effect size (RStudio, function pwr.t.test, package pwr).

Experimental protocol

MRI-compatible video cameras enabled the participants to communicate non-verbally (e.g. eye movement and facial expressions) during the experimental hyperscanning runs. Each dyad completed two experiments, one in which the patient experienced cuff pain while the clinician observed (pain MRI run), and another experiment in which the patient experienced pain while the clinician "treated" the patient's pain with remotely controlled electroacupuncture (pain/treatment MRI run).

In the ‘pain MRI’ run the patient received a series of deep pressure pain stimuli to their left lower leg. Patients received 3 moderately painful (individually calibrated to 40/100 pain) and 3 innocuous (30 mmHg pressure) standardized pressure stimuli, of 15 s duration, while the clinician observed. Prior to each pressure stimulus, both participants were shown a visual cue to indicate whether the upcoming pressure stimulus (applied to the patient) would be painful or non-painful (6-12 s jittered, the frame around the partner’s face changing color to red or green, respectively). Following each stimulus (4-10 s jittered), patients rated pain intensity (0-100 Visual Analog Scale, VAS, anchors: “No pain”, “Most pain imaginable”) using a MR compatible button box. Patients’ facial data from this scan were used to train a machine learning model which aimed to discriminate facial expression patterns associated with Pain relative to Innocuous Pressure sensation (see “Statistical analysis”).

In the ‘pain/treatment MRI', the patient received a series of 12 moderately painful (individually calibrated to 40/100 pain) pressure stimuli to their left leg. During pressure pain, the clinician applied remote electroacupuncture (EA) treatment (pseudorandomized verum, sham, and overt No-Treatment, 15 s duration). Electroacupuncture was chosen as a treatment model since it enabled practitioners to apply a pain treatment relevant to their clinical practice, in an evoked-pain, block-design experimental paradigm suitable to the fMRI hyperscanning environment. Thus, this treatment model provided a more optimized balance of ecological validity and experimental control compared to other pain therapeutic (e.g. pharmacological) models. Importantly, for verum trials, EA was applied using a minimal sub-sensory threshold current level (0.1 mA) in order to avoid unblinding patients due to any sensory feedback from electrical stimulation. This electrical current level was also unlikely to have any significant physiological effect, and pain ratings during verum EA and sham EA were statistically equivalent [2]. Prior to each pressure stimulus, both participants were shown a visual cue (6-12 s jittered, frame around the partner’s face changing color) to indicate whether-or-not the

upcoming pressure pain would be treated (green) or not treated (red) by EA, in order to evoke anticipation for treated or non-treated pain for both patients and clinicians. Correspondingly, clinicians pressed and held either a ‘treatment’ button or a different ‘no-treatment’ button (i.e. matching motor preparation and execution across trials) for the duration of applied pressure pain (blue frame). The same instructions were given to both patients and clinicians: “feel free to use your face to express how you’re feeling, as long as you keep your head as still as possible.” Thus, both patients and clinicians were equally free to express, and respond to, the other’s facial expressions. Following each stimulus, participants rated pain intensity (patients), vicarious pain (clinicians), and affect associated with the previous trial (patients and clinicians) using Visual Analog Scales.

Electroacupuncture stimulation

At the beginning of each MRI session, after the patient was positioned in the scanner, two needles (0.22 mm thick, 40 mm long MR-safe titanium, DongBang Acupuncture Inc, Boryeong, Korea) were inserted proximal to the cuff (2-3 cm depth, acupoints ST-34 and SP-10), and MRI-safe electrodes were attached to each needle. Due to hospital policy, while clinicians were encouraged to actively ‘lead’ the process, in line with their assigned role as their patient’s practitioner, actual needle penetration was performed by a staff acupuncture practitioner under direct supervision by the subject clinician, evident to the patient. The electrodes were connected to an electronic needle stimulation device (2Hz, 0.1mA, AS Super 4 Digital, Schwa-Medico, Wetzlar, Germany), controlled by the computer running the experimental protocol.

Other Materials

Cameras

Each MRI scanner was equipped with MRI-compatible cameras (Model 12M, MRC Systems GmbH, Heidelberg, Germany) attached to the table-mounted mirror, in order to enable on-line visual communication. Cameras were manually adjusted to capture the full face prior to scanning. The visual stream was projected onto a screen behind the MRI scanner bore, which the participants viewed through the table-mounted mirror. The two-way video stream (20 Hz) was transmitted over a local network (the cross-scanner delay was measured to be consistently < 40 ms) and recorded for the use of facial expression analyses.

Microphones

While verbal communication was disabled *during* scanning to avoid speech-related motion artifacts in the fMRI signal, participants were able to communicate verbally *between* different MRI scan runs. Speech was recorded using MRI-compatible optical microphones (Fibersound FOM1-MR, Micro Optics Technologies Inc., Cross Plains, WI, USA).

Software for stimulus presentation and signal synchronization

We applied in-house software (C++) for synchronization of fMRI and video signal acquisition between MRI scanners, transferring video and audio, and between-scanner network delay tracking. A laptop in each MRI scanner initiated fMRI scans using a remote trigger, and controlled the video stream, experimental visual stimuli, onset and offset of the leg pressure and EA stimuli, and recording in-scanner ratings and videos. The two laptops were connected through a Local Area Network. At the initiation of each fMRI pulse sequence, a signal from the master computer (patient MRI control room) was sent to the slave computer (clinician MRI control room). The current network delay (calculated as the mean of 10 network pings) was estimated, and the fMRI pulse sequences were then initiated locally adjusted for this network

lag, thus ensuring synchronized acquisition timing of the two fMRI time series, video streams, and experimental protocols.

Statistical analysis

Discrimination of pain states and ranking of facial feature importance

Facial AU timecourses from patients and clinicians during the pain MRI were used to train a nonlinear classifier, using XGBoost, a scalable end-to-end tree boosting algorithm which has shown state-of-the art performance in a number of diverse machine learning applications [4]. Due to the high number of decision trees trained on bootstrapped subsets of our training dataset, this method is attractive as it is inherently resistant to overfitting. The video timecourses from all available dyads was split randomly into a training (70%) and test (30%) dataset, ensuring that randomization occurred both within and between similar design blocks to avoid possible information leakage across datasets due to correlation between temporally adjacent frames. In the training set, a genetic search pipeline with an evolutionary algorithm was used to optimize hyperparameter values and their combinations[5] in a 5-fold cross-validation manner. After training, performance in the test set was assessed using an area under the curve (AUC) metric assessed from the receiver operating characteristic (ROC) curve. In order to estimate the unique contribution of each AU feature to the classification model, we calculated Shapley Additive explanations (SHAP) values [6]. At the dataset level, SHAP values allow for quantification of average feature importance in terms of total prediction power. These values, based on a game-theory framework, combine six existing methods for quantifying feature importance more consistently to human reasoning relative to previous machine learning explanatory approaches[7,8].

Directed information flow of facial expressions between patients and clinicians

We employed video streams during the pain/treatment MRI to investigate how the clinician's facial expression affected the patient's facial expression, and *vice versa,* using 'Echo-State

Granger Causality (GC)’, a GC implementation based on recurrent neural networks with minimal trainable parameter count[9,10]. GC[11] is an umbrella term for statistical methods assessing whether, given two time-series, the information contained in one (‘predicting’) series is able to improve the prediction of the future of the other (‘predicted’) series compared to employing the past of the latter only. Typically, two independent models are employed to infer the ‘predicted’ time-series, where only one model contains explicit information about the ‘predicting’ times-series. The log-ratio between the residuals of the two models commonly represents the ‘strength’ of the directed causal connection between the time-series. ‘Echo-State GC’, which we employed here, has shown superior performance in detection causality between two (possibly non-linearly) coupled dynamical variables embedded within a multivariate system[9,10].

Statistical analysis of echo-state Granger causality estimates

An important caveat inherent to Granger causality calculation between time series recorded from participants who perform the same series of tasks in a time-locked manner, is that observed causal relationships may also reflect aspects of the shared structure of the experimental paradigm rather than actual directed information flow – i.e. social interaction via facial expression communication. The null-hypothesis of no GC (i.e. zero information flow) between interacting partners’ facial expressions should therefore only be rejected if GC estimates are higher than the “pseudo GC” contributed by the shared experimental environment and structure of the experimental paradigm such as stimulus presentation timing. Thus, in order to evaluate the statistical significance of our patient-to-clinician and clinician-to-patient GC estimates, we constructed empirical null distributions for each paradigm using simulated “dyads” of subjects. Analyses using facial expression data from all possible patient-clinician combinations (‘simulated dyads’) in our dataset were generated, excluding patient-clinician dyads that actually occurred (‘real dyads’). This amounts to calculating the distribution of GC strengths

between all simulated pairs of individuals who followed the same experimental procedure but did not actually interact. GC estimate distributions for ‘real dyads’ were contrasted with these null distributions using non-parametric statistics.

MRI acquisition and preprocessing

MRI acquisition

Blood oxygen level-dependent (BOLD) brain fMRI data were collected from each participant in the dyad (Patient scanner: Siemens 3T Skyra; Clinician scanner: Siemens 3T Prisma) using a whole brain, simultaneous multi-slice, T2*-weighted gradient echo-planar imaging pulse sequence (repetition time = 1250 ms, echo time = 33 ms, flip angle = 65˚, voxel size = 2 cm isotropic, number of slices = 75, Multiband acceleration factor = 5, 624 volumes split into 2 consecutive scan runs). Since the Siemens 3T Skyra has a slightly larger bore space compared to the Prisma, we decided to use this scanner for the fibromyalgia patient group, in order to maximize scanner comfort. Furthermore, keeping a designated “patient scanner” and “clinician scanner”, rather than randomizing scanner assignment between dyads, improved protocol consistency within patient and clinician groups and facilitated the setup of our hyperscanning infrastructure.

A high-resolution structural volume (multi-echo MPRAGE) was collected to facilitate anatomical localization and spatial registration of individual BOLD fMRI volumes to standard space (Montreal Neurological Institute, MNI, 152) (repetition time = 2530 ms, echo time = 1.69 ms, flip angle = 7˚, voxel size = 1 mm isotropic). Importantly, to enable a full-face view of each participant for better facial expression tracking by the facial expression digitization software (see below), while still allowing for full brain fMRI data acquisition, we combined the occipital/bottom portion of a 64 Channel head coil with a flex coil (4 channel) attached to the forehead.

fMRI preprocessing

Preprocessing of individual fMRI datasets was carried out using tools from FMRIB's Software Library (FSL, v6.0.0; www.fmrib.ox.ac.uk/fsl), and included the following steps: slice-timing correction, motion correction (MCFLIRT)[12], correction of spatial inhomogeneity (TOPUP)[13,14], nonbrain tissue removal (BET)[15], spatial smoothing (full width at half maximum = 4mm), temporal high-pass filtering (f=0.011 Hz as computed by FSL's cutoffcalc), and grand-mean intensity normalization by a single multiplicative factor. For each subject, both runs were realigned (6 degrees of freedom) to a common reference space (7th volume of the first run) before the first-level GLM analyses. The transformation matrix for registration between functional and high-resolution anatomical volumes was calculated using Boundary Based Registration (bbregister, Freesurfer, v6.0.0[16]). Two participants had one of their two fMRI pain/treatment MRI runs excluded from analysis due to excessive head motion, based on the following exclusion criteria: 1) >2° frame-by-frame head rotation in any direction, and 2) >2 mm frame-by-frame displacement. After excluding these data, mean head rotation was 0.05±0.02 (Mean±SD) and mean frame-by-frame displacement was 0.13±0.05 mm. An unpaired t-test indicated higher frame-by-frame displacement for patients (0.15±0.05) relative to clinicians (0.11±0.04, t=4.32, P<0.001), but there was no significant group difference for rotation (t=1.74, P=0.09). For registration from structural to standard space (MNI152), we used FSL's Linear registration tool (FLIRT, 12 degrees of freedom)[12,17], followed by FSL's non-linear registration tool (FNIRT)[18]. All single-subject analyses were performed in functional space, and then registered to MNI152 standard space before dyadic and group analyses.

References

1 Wolfe F, Häuser W. Fibromyalgia diagnosis and diagnostic criteria. *Ann Med* 2011; **43**: 495–502.

2 Ellingsen D-M, Isenburg K, Jung C, Lee J, Gerber J, Mawla I *et al.* Dynamic brain-to-brain concordance and behavioral mirroring as a mechanism of the patient-clinician interaction. *Science Advances* 2020; **6**: eabc1304.

3 Jensen KB, Petrovic P, Kerr CE, Kirsch I, Raicek J, Cheetham A *et al.* Sharing pain and relief: neural correlates of physicians during treatment of patients. *Mol Psychiatry* 2014; **19**: 392–398.

4 Olson RS, La Cava W, Mustahsan Z, Varik A, Moore JH. Data-driven Advice for Applying Machine Learning to Bioinformatics Problems. *arXiv:170805070 [cs, q-bio, stat]* 2018.http://arxiv.org/abs/1708.05070 (accessed 16 Mar2021).

5 Le TT, Fu W, Moore JH. Scaling tree-based automated machine learning to biomedical big data with a feature set selector. *Bioinformatics* 2020; **36**: 250–256.

6 Štrumbelj E, Kononenko I. Explaining prediction models and individual predictions with feature contributions. *Knowl Inf Syst* 2014; **41**: 647–665.

7 Lundberg SM, Lee S-I. A Unified Approach to Interpreting Model Predictions. *Advances in Neural Information Processing Systems* 2017; **30**.https://proceedings.neurips.cc/paper/2017/hash/8a20a8621978632d76c43dfd28b67767-Abstract.html (accessed 12 Mar2021).

8 Lundberg SM, Erion G, Chen H, DeGrave A, Prutkin JM, Nair B *et al.* From local explanations to global understanding with explainable AI for trees. *Nature Machine Intelligence* 2020; **2**: 56–67.

9 Duggento A, Guerrisi M, Toschi N. Recurrent neural networks for reconstructing complex directed brain connectivity. In: *2019 41st Annual International Conference of the IEEE Engineering in Medicine and Biology Society (EMBC)*. 2019, pp 6418–6421.

10 Duggento A, Guerrisi M, Toschi N. Echo State Network models for nonlinear Granger causality. *bioRxiv* 2019; : 651679.

11 Granger CWJ. Investigating Causal Relations by Econometric Models and Cross-spectral Methods. *Econometrica* 1969; **37**: 424–438.

12 Jenkinson M, Bannister P, Brady M, Smith S. Improved optimization for the robust and accurate linear registration and motion correction of brain images. *Neuroimage* 2002; **17**: 825–841.

13 Andersson JLR, Skare S, Ashburner J. How to correct susceptibility distortions in spin-echo echo-planar images: application to diffusion tensor imaging. *Neuroimage* 2003; **20**: 870–888.

14 Smith SM, Jenkinson M, Woolrich MW, Beckmann CF, Behrens TEJ, Johansen-Berg H *et al.* Advances in functional and structural MR image analysis and implementation as FSL. *Neuroimage* 2004; **23**: S208–S219.

15 Smith SM. Fast robust automated brain extraction. *Hum Brain Mapp* 2002; **17**: 143–55.

16 Greve DN, Fischl B. Accurate and robust brain image alignment using boundary-based registration. *Neuroimage* 2009; **48**: 63–72.

17 Jenkinson M, Smith S. A global optimisation method for robust affine registration of brain images. *Med Image Anal* 2001; **5**: 143–56.

18 Andersson J, Jenkinson M, Smith S. Non-linear registration, aka spatial normalisation, FMRIB technical report TR07JA2. 2010.

Supplementary tables

Table S1: Facial Action Units (AU) extracted from in-scanner facial expression videos

Description	FACS AU	Facial muscle(s)
Inner brow raiser	1	Frontalis (pars medialis)
Outer brow raiser	2	Frontalis (pars lateralis)
Brow furrowing	4	Depressor glabellae, Depressor supercilii, Currogator supercilii
Cheek raiser	6	Orbicularis oculi (pars obitalis)
Smile	6/12	Orbitularis oculi, Zygomaticus major
Lid tightener	7	Orbicularis oculi, (pars palpebralis)
Nose wrinkler	9	Levator labii superioris alaeque nasi
Upper lip raiser	10	Levator labii superioris, Caput infraorbitalis
Dimpler	14	Buccinator
Lip corner depressor	15	Depressor anguli oris
Chin raiser	17	Mentalis
Lip pucker	18	Incisivii labii superioris, Invisivii labii inferioris
Lip stretcher	20	Risorius, platysma
Lip pressor	24	Orbicularis oris
Jaw drop	26	Masseter, Temporalis, Internal Pterygoid
Mouth open	25/27	Depressor labii, Orbicularis oris, Pterygoid, Digastricus
Lip suck	28	Orbicularis oris
Eye closure	43	Levator palpebrae superioris
Smirk	N/A	Zygomaticus major
Eye widening	N/A	Orbicularis oculi, Levator palpebrae superioris

Supplementary Figures

Figure S1:

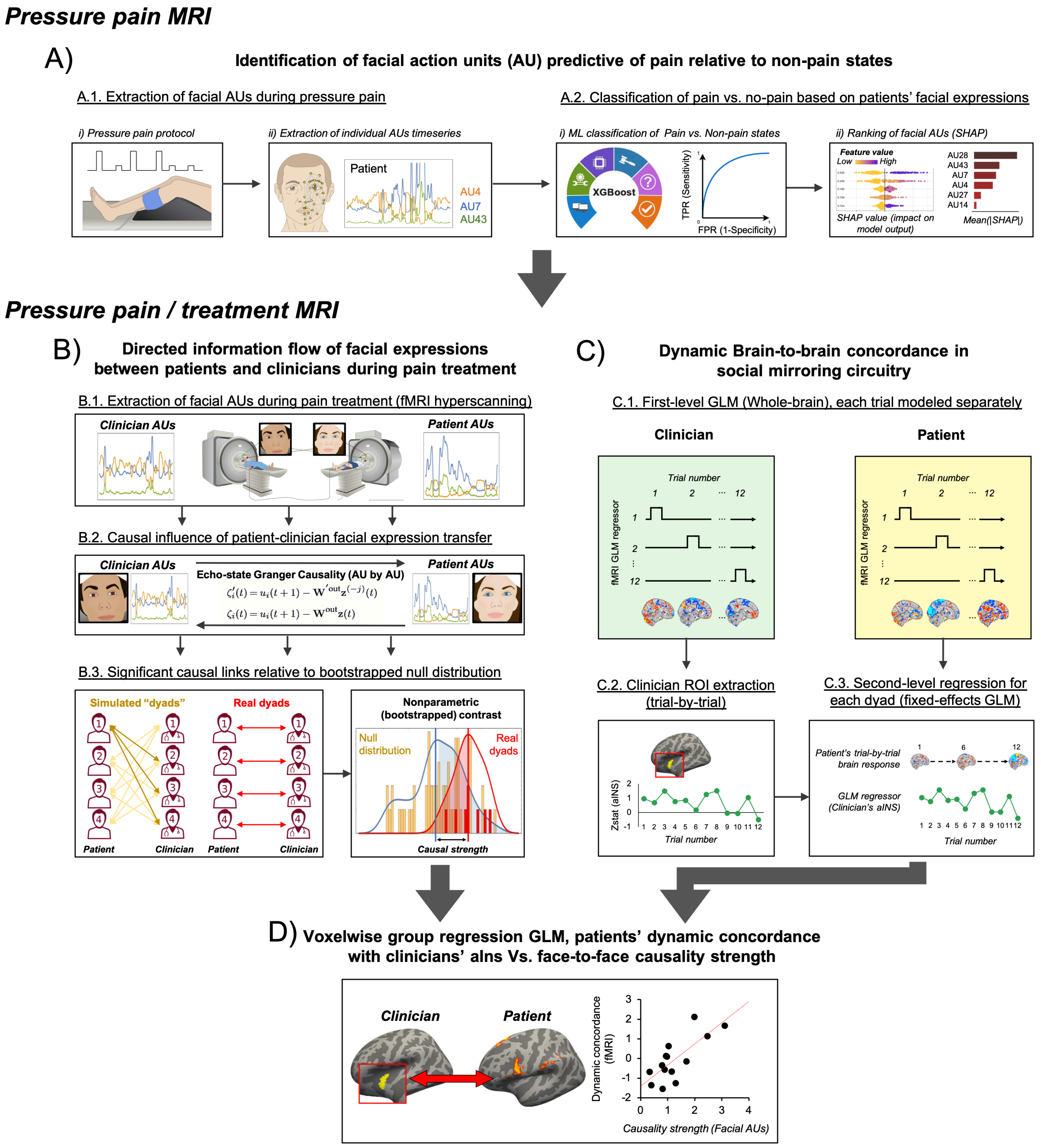

Figure S1: *Summary of the analysis approach. A.1: In order to identify the features of patients' facial expressions that were indicative of pain, we first applied automated extraction of facial AUs (n=20 AUs) using video recordings of patients' faces during moderate cuff-evoked leg pressure pain and non-painful cuff pressure. A.2: We then used these AU timeseries to train a*

nonlinear classifier (XGBoost) to discriminate between moderately painful and non-painful leg pressure. Next, we used an explainability technique (SHAP) to rank all AUs according to their unique contribution to the classification outcome. This ranking was used to guide subsequent analyses investigating directed information flow between pain-related facial features. B.1: Similarly, we extracted AUs from the subsequent Pain/Treatment MRI, in which patient-clinician dyads interacted as part of a pain treatment protocol. B.2. AU timeseries from patients and clinicians were used to estimate multivariate, nonlinear, neural-network based causality matrices that quantify directional influence (i.e. information transfer) between patients' and clinicians' facial expressions. B.3: Next, we estimated "pseudo-causality" between simulated "dyads", i.e. all possible patient-clinician pairs who did not interact. This step was done in order to generate a fully data-driven null distribution of causality estimates when there was no actual social interaction, thus controlling for non-interactive aspects of the experimental protocol (e.g. shared timing of visual cues and pain stimulus presentation). This null distribution was contrasted with the distribution of causality strengths derived from real dyads. P-values were corrected for multiple comparisons using False Discovery Rate across the entire matrix of causal links (20×20=400 causal links for each direction – patient-to-clinician and clinician-to-patient). C.1. In order to calculate patient-clinician concordance in brain activity, we first conducted a first-level GLM for each individual's preprocessed fMRI timeseries, with each trial as a separate regressor. C.2. Next, trial-by-trial mean Z-values were extracted from a priori Regions of Interest (e.g. aINS) implicated in social mirroring and theory-of-mind. C.3. These values were applied in a second-level fixed effects GLM of the partner's temporally synchronized fMRI data, to calculate voxelwise estimates of cross-brain dynamic concordance for each dyad. D) Finally, the aggregated causal influence of patient-to-clinician information flow were used in a voxel-wise group regression GLM in order to investigate the association of brain-to-brain concordance patterns with directed facial communication dynamics.

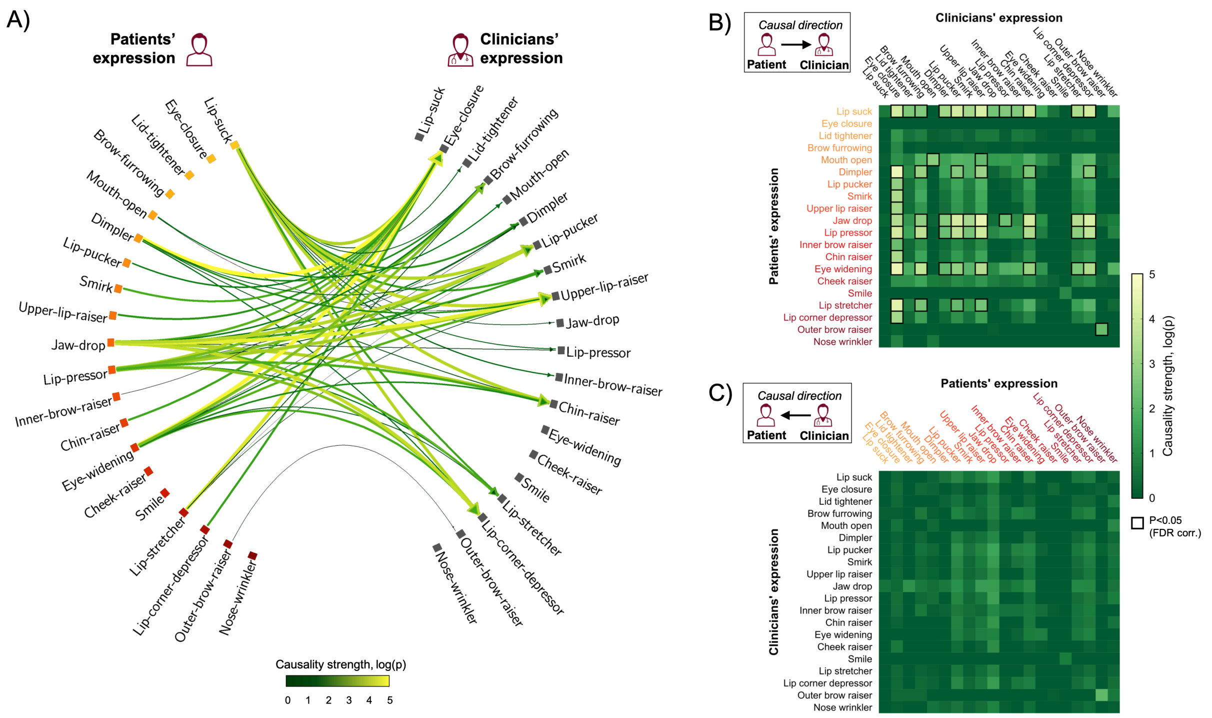

Figure S2: *Median (across dyads) Granger causality strength between patients' and clinicians' facial action units (AUs) during pain and treatment/no-treatment. (A) Significant causal links between Patients' AUs and Clinicians' AUs, surviving False Discovery Rate (FDR) correction for multiple comparisons, are shown with causal direction indicated by arrowheads and causality strength (log(p)) indicated by color and thickness. AUs are ordered according to SHAP values (the most influential AU at the top row) reflecting AU association with patients' pain perception – i.e. importance in discriminating pain from innocuous pressure when no treatment was provided (i.e. bright yellow indicates a large, whereas dark red indicates a small, contribution to machine learning-based prediction of patients' pain versus innocuous pressure, corresponding to the color shading in Fig. 2). Matrices (right) show causal links from the patient to the clinician (B) and from the clinician to the patient (C). Same as for the connectogram (A), AUs are ordered according to their ranked contribution to the independent classification of pain for non-pain states (bright yellow indicates large, while dark red indicates small contribution). Each cell in the matrices shows the corresponding Granger Causality strength. The causal links that survived FDR correction for multiple comparisons across all*

possible matrix cells are highlighted by black rectangles. Patients' 'lip suck', which showed the strongest unique contribution to the discrimination of pain states, was also the AU that influenced the dynamics of the largest number of facial AU in the clinician when treating evoked cuff pressure pain (B). No significant causal links were found in the opposite direction - i.e. between clinicians' facial AUs and patients' AUs (C).
